# Clinical outcomes of children and adolescents with sickle cell disease and COVID-19 infection: A year in review at a metropolitan tertiary pediatric hospital

**DOI:** 10.3389/fmed.2023.987194

**Published:** 2023-02-17

**Authors:** Olufunke Y. Martin, Deepika S. Darbari, Stefanie Margulies, Robert S. Nickel, Alexis Leonard, Barbara Speller-Brown, Brenda Martin, John R. Barber, Jennifer Webb, Suvankar Majumdar, Matthew P. Sharron, Andrew D. Campbell

**Affiliations:** ^1^Center for Cancer and Blood Disorders, Division of Hematology, Children’s National Hospital, Washington, DC, United States; ^2^George Washington University School of Medicine and Health Sciences, Children’s National Hospital, Washington, DC, United States; ^3^Department of Biostatistics and Study Methodology, Children’s National Hospital, Washington, DC, United States; ^4^Department of Pediatrics, The George Washington University School of Medicine, Washington, DC, United States

**Keywords:** sickle cell disease (SCD), COVID-19, SARS-CoV-2, pediatrics–children, morbidity, mortality

## Abstract

**Background:**

COVID-19 was declared a global pandemic in March 2020. Early reports were primarily in adults, and sickle cell disease (SCD) was classified as a risk factor for severe COVID-19 disease. However, there are a limited number of primarily multi-center studies reporting on the clinical course of pediatric patients with SCD and COVID-19.

**Methods:**

We conducted an observational study of all patients with SCD diagnosed with COVID-19 at our institution between March 31, 2020, and February 12, 2021. Demographic and clinical characteristics of this group were collected by retrospective chart review.

**Results:**

A total of 55 patients were studied, including 38 children and 17 adolescents. Demographics, acute COVID-19 clinical presentation, respiratory support, laboratory findings, healthcare utilization, and SCD modifying therapies were comparable between the children and adolescents. Seventy-three percent (*N* = 40) of all patients required emergency department care or hospitalization. While 47% (*N* = 26) were hospitalized, only 5% (*N* = 3) of all patients required intensive care unit admission. Patients frequently had concurrent vaso-occlusive pain crisis (VOC) (*N* = 17, 43%) and acute chest syndrome (ACS) (*N* = 14, 35%). Those with ACS or an oxygen requirement had significantly higher white blood cell count, lower nadir hemoglobin, and higher D-dimers, supporting a pro-inflammatory and coagulopathic picture. Non-hospitalized patients were more likely to be on hydroxyurea than hospitalized patients (79 vs. 50%, *p* = 0.023).

**Conclusion:**

Children and adolescent patients with SCD and acute COVID-19 often present with ACS and VOC pain requiring hospital-level care. Hydroxyurea treatment appears to be protective. We observed no mortality despite variable morbidity.

## Introduction

COVID-19 (SARS-CoV-2) was declared a global pandemic by the World Health Organization in January 2019 leading to over 500 million confirmed cases and greater than >6 million deaths worldwide (1). During the earlier phases of the pandemic, higher morbidity and mortality were concentrated within the elderly and patients with underlying chronic conditions, including diabetes, obesity, and sickle cell disease (SCD) (2). In children, the majority presented either asymptomatic or with mild symptoms (3). A study of 277,285 school-aged children with laboratory-confirmed COVID-19 in the United States (U.S.) from March 2020-September 2020 reported only 1.2% were hospitalized and 40% were asymptomatic (3). However, children with an underlying medical condition accounted for 16% of hospitalizations, 27% of Intensive Care Unit (ICU) admissions, and 28% of the mortality (3). A multi-center study stratifying 2,293 hospitalized SARS-CoV-2 positive children by specific underlying conditions revealed those with chronic lung disease and neurologic disorders were associated with highest risk for severe COVID-19 infection (4). Children’s National Hospital (CNH) also confirmed this association reporting higher hospitalization rates within their cohort of 177 SARS-CoV-2 positive children and adolescents with an underlying medical condition (5).

Sickle cell disease has been identified as a risk factor for severe COVID-19 disease. The effects of COVID-19 on the clinical course of SCD are emerging most notably through case reports, multi-center and single-center experiences, and through the international SECURE Sickle Cell COVID-19 registry (6–8). A published report of the 750 SCD COVID-19 cases from the SECURE registry revealed that SARS-CoV-2 severely affects SCD with an increase in morbidity and a higher case fatality rate than the general population (7). Sixty-nine percent of adults and 40% of children were hospitalized, in addition to 5.8 and 8.8% admitted to the intensive care unit, respectively (7). With a reported mortality rate of 2.5% from the SECURE registry, COVID-19 SCD patients suffered higher hospitalizations and case-fatality rates than those of similar ages within the US population (7). To comprehensively evaluate the impact of COVID-19 on pediatric patients, we conducted this study describing our one-year experience caring for a large number of patients with SCD and COVID-19 early in the COVID-19 pandemic.

## Methods

This was a single-center, observational, prospective cohort study describing the clinical course of COVID-19 in pediatric (age <18 years) and adolescents (age 18–21 years) SCD patients. CNH is a free-standing 323-bed quaternary academic medical center that serves over 223,000 unique patients primarily from Maryland (59%) and Washington DC (23%), including over 1,500 pediatric and adolescent patients with sickle cell disease through outreach with local hospitals and clinics. We started our CNH Sickle Cell Disease COVID-19 Registry in March 2020 to collect real-time clinical information in SCD patients aged 0–21 years old (y/o) presenting with polymerase chain reaction (PCR) confirmed SARS-CoV-2 infection either in their community or at our hospital and associated clinics. As adapted by the Sickle Cell SECURE registry, we classified patients by level of severity (five levels) during their COVID-19 clinical course (6).

The study was approved by the CNH Institutional Review Board. We collected demographics, clinical characteristics, presenting symptoms, management, treatment, and clinical outcomes prospectively in patients with SCD and COVID-19 infection admitted between March 2020 and February 2021. In April 2020, we implemented standardized COVID-19 management and treatment guidelines for patients with SCD treated at our institution. As the pandemic progressed and updated treatment guidelines were published there was an evolution of our outpatient and inpatient guidelines for screening of SCD patients for COVID-19, laboratory, and clinical guidelines for suspected versus confirmed COVID-19, and eligibility criteria for monoclonal antibody therapy, antiviral treatment, and prophylactic anticoagulation (9–11). Inpatient prophylactic anticoagulation was initiated at the time of positive SARS-CoV-2 PCR and continued throughout hospitalization until 30 days post discharge with a telehealth visit with a hematology provider at 2 weeks.

### Statistical analysis

Analysis was performed using descriptive statistics with categorical variables presented as proportions and continuous variables presented with their median and interquartile ranges. Differences were tested using the Chi-Square test or Fisher’s Exact test for categorical variables and the Wilcoxon Rank Sum Test for continuous variables. All tests were two-sided and the values of *p* < 0.05 were considered statistically significant. The statistical analysis was done using SAS V9.4 (Cary, NC, USA).

## Results

### Demographics and clinical characteristics

Fifty-five patients with SCD had PCR-confirmed SARS-CoV-2 during the study time period, representing ∼3.7% of our patient population, of which 69% were children (*N* = 38, <18 years) versus 31% were adolescents (*N* = 17, 18–21 years) (Table 1). The mean age was 11.6 years for the study population with mean ages of 8.3 and 19.2 years in children and adolescents, respectively. Gender was evenly matched with 51% females and 49% males. Hemoglobin SS (Hgb SS) was the most common genotype with 74% of the cases (79% of pediatrics, 65% of adolescents) followed by Hemoglobin SC (Hgb SC) (15%) and Sickle -Beta Zero Thalassemia (Hgb Sβ0 Thal) SCD (11%). Hydroxyurea (HU) was the most common disease-modifying therapy in 66% of children and 65% of adolescents; 7% were on chronic blood transfusions, 5% on crizanlizumab, and 4% on voxelotor (Table 1). All patients receiving crizanlizumab or voxelotor were also on HU.

**TABLE 1:** Demographics and baseline characteristics.

	All patients *N* = 55	Children <18 y/o (*N* = 38, 69%)	Adolescents ≥18 y/o (*N* = 17, 31%)
Age mean (SD)	11.6 (6.7)	8.3 (5.2)	19.2 (1.2)
Age, median (IQR)	13.0 (5.0–18.0)	7.0 (4.0–14.0)	19 (18.0–20.0)
**Gender *N* (%)**			
Female	28 (51)	21 (55)	7 (41)
Male	27 (49)	17 (45)	10 (59)
Genotype (*N* = 55) *N* (%)*			
Hgb SS	41 (74)	30 (79)	11 (65)
Hgb SC	8 (15)	4 (11)	4 (24)
Hgb Sβ0 Thal	6 (11)	4 (11)	2 (12)
**Disease modifying therapies *N* (%)**			
Chronic blood transfusion	4 (7)	4 (11)	0 (0)
Hydroxyurea	36 (65)	25 (66)	11 (65)
Crizanlizumab	3 (5)	0 (0)	3 (18)
Voxelotor	2 (4)	1 (3)	1 (6)

*Numbers may not add up to 100% due to rounding.

Hgb, hemoglobin; Sβ0 Thal, sickle beta zero thalassemia; ED, emergency department; Hosp, hospitalization; ICU, intensive care unit.

### Health care utilization

**FIGURE 1:**
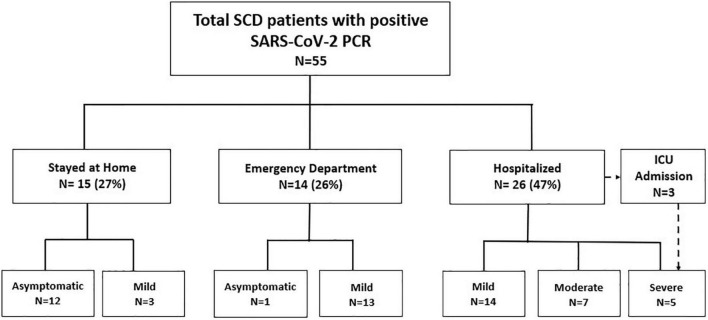
Healthcare utilization.

Twenty-seven percent (*N* = 15) of SCD patients with positive SARS-CoV-2 PCRs remained at home and were tested in an outpatient or community setting for mild symptoms or asymptomatic screening (Figure 1). The remaining 73% (*N* = 40) received emergency department (ED) care or were hospitalized (Figure 1). Twenty-six percent (*N* = 14) were discharged from the ED, of which the majority were children (*N* = 10). Approximately half (47%, *N* = 26) of the SCD patients with COVID-19 were hospitalized (45% of children, 53% of adolescents). Only 3 patients (5% of all patients) required Intensive Care Unit (ICU) level care for management of multi-lobar ACS and hypoxia requiring BiPAP (Supplementary Tables 3, 5). The median length of hospitalization was 5 days (4 days for children, 6 days for adolescents) (Table 2).

**TABLE 2:** Clinical presentation and characteristics of hospitalized and ED patients.

*N* (%)	All patients *N* = 40	Children, <18 y/o (*N* = 27)	Adolescents ≥18 y/o (*N* = 13)
Fever ≥38°C during hospitalization or ED visit	24 (60)	19 (70)	5 (38)
Oxygen saturation <95%	15 (38)	6 (22)	9 (69)
**Sickle cell disease presentation**			
Vaso-occlusive crisis (Pain)	17 (43)	11 (41)	6 (46)
Acute chest syndrome	14 (35)	7 (26)	7 (54)
Splenic sequestration	1 (3)	1 (4)	0 (0)
Venous thromboembolism	1 (3)	0 (0)	1 (8)
Length of stay** (day) median (IQR)	5 (3–6)	4 (2–5)	6 (4–6)

**Only hospitalized patients (*N* = 26) included.

ED, emergency department.

### COVID-19 exposures and clinical presentation

**FIGURE 2:**
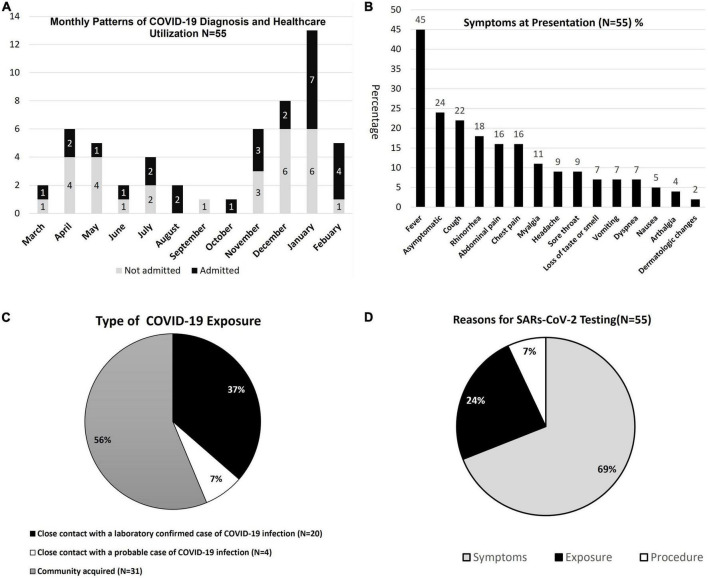
**(A–D)** COVID-19 admission patterns, symptoms, exposures, and testing.

Figure 2A shows the monthly patterns of COVID-19 cases in our SCD patients from March 2020 through February 2021. There were 3 peaks of COVID-19 cases represented in spring 2020 (*N* = 11 April-May 2020), summer 2020 (*N* = 6 July-August 2020), and winter 2020–21 (*N* = 21 December 2020-January 2021). Classification of symptoms at presentation (Figure 2B) and type of exposure (Figure 2C) were based on classifications from the SECURE registry. Fever (45%) was the most common presenting symptom in our SCD patients with COVID-19. Cough (22%), rhinorrhea (18%), abdominal pain (16%), chest pain (16%), sore throat (9%), and myalgias (11%) were among the other symptoms (Figure 2B). Only 7% percent reported a loss of taste or smell. Conversely, 24% of patients were asymptomatic at time of positive SARS-CoV-2 PCR. Among the 40 patients with SCD and COVID-19 who were hospitalized or had ED visits, 60% (*N* = 24) presented with fever, 43% (*N* = 17) with vaso-occlusive pain crisis (VOC), 35% (*N* = 14) with acute chest syndrome (ACS), 3% (*N* = 1) with splenic sequestration and 3% (*N* = 1) with venous thromboembolism (Table 2).

Exposure to COVID-19 was primarily community-acquired (56%, *N* = 31), followed by close contact of laboratory-confirmed COVID-19 (37%, *N* = 20), and close contact of a probable case of COVID-19 in 7% (Figure 1C). Sixty-nine percent (*N* = 38) of patients reported reasons for COVID-19 testing were predominately for symptoms, 24% (*N* = 13) were tested for exposure, and 7% (*N* = 4) on pre-anesthesia assessment for procedures or diagnostic studies (Figure 2D).

### Clinical severity and laboratory presentation

Twenty-four percent were asymptomatic (severity), while 54% experienced mild severity, primarily with an upper respiratory infection or gastrointestinal symptoms during their COVID-19 infection (Table 3). Moderate severity defined by pneumonia without hypoxia was exhibited in 13%, and severe presentation [dyspnea and hypoxia, oxygen saturation (02 sat) <92%] occurred in 9% of COVID-19 SCD patients (Table 3). A complete blood cell count (CBC) in 43 patients revealed a median white blood cell (WBC) count 10.4 × 10^3^/μL (7.6–14.9 IQR), hemoglobin (hgb) 8.4 g/dL (7.3–10.2 IQR), and platelet count 277 × 10^3^/μL (190–380 IQR) (Supplementary Table 1). Elevated median d-dimers of 1.7 μg/mL (0.5–4.7 IQR) and CRP of 2.0 mg/dL (0.4–6.2 IQR) were found within a subset of SCD patients with COVID-19 (Supplementary Table 1).

**TABLE 3:** Severity of sickle cell disease during+COVID-19 period*.

Level of severity	Definition	N	*%*
Asymptomatic	No clinical signs or symptoms during the positive COVID-19 period	13	24%
Mild	Symptoms of acute upper respiratory tract infection, including fever, fatigue, myalgia, cough, sore throat, runny nose, and sneezing or gastrointestinal symptoms or digestive symptoms such as nausea, vomiting, abdominal pain and diarrhea	30	54%
Moderate	Pneumonia, with or without clinical symptoms, no hypoxia	7	13%
Severe	Early respiratory symptoms or gastrointestinal symptoms followed by dyspnea and hypoxia (oxygen saturation less than 92%)	5	9%
Critical	Acute respiratory distress syndrome, respiratory failure, encephalopathy, shock, coagulopathy, multiorgan impairment (lung, heart, kidney, brain) that may be life threatening	0	0%

*Adapted from the SECURE registry (7).

### COVID-19 and sickle cell supportive treatment

Fifty-one percent of SCD patients received antibiotics, including ceftriaxone (51%) and azithromycin (25%) [Data not shown]. Only 6 SCD patients were treated with Remdesivir and 1 with convalescent plasma as early in the pandemic inpatient use was restricted to those with an oxygen requirement and higher clinical severity. Consequently, all patients that received these therapies carried a diagnosis of ACS. Blood transfusions were given to 29% (*N* = 16), which represented 61.5% of admitted patients. One patient received both simple and exchange transfusions. Twenty-six percent (*N* = 17) of hospitalized patients with SCD and COVID-19 received anticoagulant thromboprophylaxis with either enoxaparin or rivaroxaban according to the CNH COVID-19 anticoagulation treatment protocol. Inhaled or nebulized albuterol and budesonide were given to 37 and 22% of COVID-19 SCD patients, respectively. No patients received oral steroids.

### Acute chest syndrome (ACS) vs. no acute chest syndrome

Thirty-five percent of SCD patients with COVID-19 were diagnosed with ACS and 92% of those had multi-lobar infiltrates on CXR. Lower 02 Sat <95% (86% vs. 8% *p* < 0.001), higher WBC count (14.1 vs. 8.7 × 10^3^/μL, *p* = 0.033), lower hgb nadir (6.8 and 9.6 g/dL, *p* = 0.064), and elevated D-dimers (3.6 vs. 0.48 μg/mL, *p* = < 0.001) were significantly associated with ACS patients (Supplementary Table 2). One patient required an automated exchange blood transfusion.

### Oxygen requirement vs. no oxygen requirement

All SCD patients with COVID-19 requiring supplemental oxygen received a blood transfusion compared to only 38% of those who were not on supplemental oxygen (*p* < 0.001) (Supplementary Table 3). Additionally, 90% of patients with oxygen requirement showed pneumonia on CXR (all with multi-lobar infiltrates) vs. only 14% who were not on oxygen (Supplementary Table 4). One patient exhibited bilateral pleural effusions but without pulmonary infiltrates. Higher WBC count (8.8 vs. 14 × 10^3^/μ, *p* = 0.002), lower hgb (9.0 vs. 7.1 g/dL, *p* = 0.002), higher D-Dimer (0.8 vs. 4.1 μg/mL, *p* = 0.002), higher fibrinogen (270 vs. 601 mg/dL, *p* = 0.013), higher prothrombin time (14.5 vs. 16 s, *p* = 0.006) were all associated with supplemental oxygen requirement (Supplementary Table 4).

### Potential risk/protective factors for severe COVID-19

#### Hospitalized vs. non-hospitalized patients

Hemoglobin SS and Hgb SC showed similar proportion of patients hospitalized vs. non-hospitalized with COVID-19 (Table 4). Fever (69% vs. 24%, *p* = 0.001), VOC pain crisis (50% vs. 24%, *p* = 0.047), and ACS (54% vs. 0%, *p* < 0.001) were more common in hospitalized SCD patients (Table 4). Hematologic studies including CBC, coagulation labs, inflammatory markers were not significantly different between hospitalized and non-hospitalized patients (Supplementary Table 1).

**TABLE 4:** Sickle cell disease (SCD) COVID-19 patients non-hospitalized vs. hospitalized.

	Non-hospitalized (*N* = 29)	Hospitalized (*N* = 26)	*P-value
Age median (IQR)	11 (6–18)	14 (5–18)	0.473
**Sex (N,%)**			
Male	13 (45%)	14 (54%)	0.504
Female	16 (55%)	12 (46%)	
**Sickle cell genotype *N* (%)**			
Hgb SS	22 (76%)	19 (73%)	0.553
Hgb SC	5 (17%)	3 (12%)	
Hgb Sβ0 Thal	2 (7%)	4 (15%)	
Hydroxyurea use	23 (79%)	13 (50%)	0.023
Oxygen saturation *N* (%) <95%	2 (10%) *N* = 20	13 (62%) *N* = 21	<0.001
**Symptoms *N* (%)**			
Fever	7 (24%)	18 (69%)	<0.001
**Sickle cell disease presentation *N* (%)**			
Vaso-occlusive crisis (any)	7 (24%)	13 (50%)	0.047
Acute chest syndrome	0 (0%)	14 (54%)	<0.001

*P-value: Non-hospitalization vs. hospitalization. Hgb, hemoglobin; Sβ0 Thal, sickle beta zero thalassemia.

#### Children vs. adolescent patients

The demographics and clinical presentation between our SCD children and adolescent patients with COVID-19 were similar overall. Symptoms of COVID-19, SCD genotype, HU use, voxelotor use, sickle cell symptoms, hospitalization rate, length of stay, respiratory support, oxygen requirement, blood transfusions, ACS episodes, CBC, inflammatory markers were not significantly different in children vs. adolescents in our cohort. However, adolescents were more likely to be on crizanlizumab treatment (0% vs. 18%, *p* = 0.026) and have an elevated D-Dimer (1.0 vs. 4.0 μg/mL, *p* = 0.012) on laboratory studies (Supplementary Table 5).

### Hydroxyurea

Thirty-six patients (65%) were on HU. The median age on HU was 14 y/o versus 9.5 y/o not on HU (*p* = 0.166). Seventy-six percent (*n* = 31) of patients with HbSS and 33% (*n* = 2) of HbSB0 patients were on HU while the remaining HU patients were Hgb SC (38%, *n* = 3) (*p* = 0.017). No significant differences in length of stay, the prevalence of ACS, respiratory support, oxygen requirement, blood transfusions, CBC, coagulation labs, and inflammatory markers were found between those on HU at baseline, compared to those not on HU. However, patients taking HU were less likely to be hospitalized than those not taking HU (79%% vs. 50%, *p* = 0.023) (Table 4). Furthermore, 2 of the 3 patients requiring ICU level care were not on HU.

## Discussion

Our study is one of a few pediatric comprehensive reports on the 1-year experience of patients with SCD and COVID-19 during the pandemic. We report that SCD remains a significant risk factor for morbidity in patients with COVID-19 disease. Specifically, the majority of pediatric and adolescent patients with SCD and COVID-19 sought medical care and nearly half required hospitalization. Patients not on HU were more likely to be hospitalized. While VOC, fever, and ACS were the most common presenting symptoms in those evaluated in the hospital setting, overall COVID-19 clinical severity was mild in most patients. COVID-19 infection rates in our SCD cohort mirrored the incidence of reported COVID-19 infection in our region (5). Comparatively, clinical severity reported from the SECURE registry showed that 18% of SCD patients with COVID-19 were asymptomatic, 56% had mild disease severity, 13% had moderate disease severity, 11% had severe disease severity, and 2% had critical disease severity (7). VOC was the most common SCD-specific presenting symptom and mechanical ventilation was required in 2.4% of their cohort.

One of the largest (non-SECURE registry) case series of COVID-19 SCD cases to date was the French experience which reported 83 hospitalized SCD patients infected by SARS-CoV-2 (12). They included 24 different centers with patients ranging from 3 months to 74 y/o (12). Fifty-four percent of patients presented with VOC and 28% with ACS. Among the 20% (*N* = 17) who were admitted to the ICU, 53% (*N* = 9) required Mechanical Ventilation, and 12% (*N* = 2) required extracorporeal membrane oxygenation (12). Previously non-SECURE registry published reports on COVID-19 SCD patients have primarily been adults, and reports in pediatrics have been lacking in larger numbers (13, 14). A study from the US Peds COVID-19 registry, evaluated 27 pediatric patients with SCD and demonstrated increased morbidity and hospitalization rates although no patients required ICU level care (15). Minniti et al. published the largest US study (4 metropolitan areas) to date reporting on cases but included only 9 pediatric patients confirming a higher mortality rate in adults and those with pre-existing end-organ damage (13).

As compared to the general US population, our rate of hospitalization (47%) and need for Intensive Care-ICU (5%) is much higher than that reported (2.5% admission, 0.8% ICU) for children and adolescents (ages 0–24 years) (16) but in line with what has previously been reported for pediatric patients with SCD in a report of pediatric hematology/oncology patients in Texas (17) (47% hospitalization, 7% ICU) as well as for 0–18 years old in the SECURE registry (40.1% hospitalization, 5.8% ICU) (7). We had no mortality or need for mechanical ventilation or extracorporeal membrane oxygenation therapies despite sickle cell patients being relatively immunosuppressed. Our absence of mortality is also consistent with an investigation using the TriNetX database which demonstrated that even though patients with SCD have significantly higher rates of hospitalization, ACS, and VOC due to COVID-19, they do not have increased rates of mortality when a 1:1 propensity score matched comparison to the non-SCD Black population with COVID-19 was performed (18).

Of patients evaluated in a hospital setting (ED or inpatient), VOC (50%), and fever (45%) were the most common symptoms seen, and these same symptoms along with ACS were statistically more likely to lead to hospitalization. Patients with ACS and/or an oxygen requirement had significantly higher WBC count, lower nadir hemoglobin, and higher D-dimers. The D-dimer finding, though hard to interpret given its fluctuation with sickling of red blood cells (RBCs), is interesting as it has been shown to be an independent risk factor for death in both sickle cell and non-sickle cell patients with COVID (6, 13, 19). Also notable is that thromboembolism occurred in only a single adolescent patient (2%), despite there being an association between thromboembolism and both SCD (20) and COVID-19 (21). Some of this can possibly be explained by an early recognition of the risk of clotting, and proactive adherence to prophylactic anticoagulation at our institution without major or clinically relevant non-major bleeding. Hydroxyurea was the most common disease-modifying therapy which has been shown to decrease ACS and need for RBC transfusion in pediatric patients with SCD (22, 23). Two-thirds of our cohort was on HU and were less likely to be hospitalized than patients not receiving it confirming the protective effect of HU. This HU usage level was higher than both the SECURE registry (56% for children 0–18 y/o) (7) and the French experience (46% of total, 33% in children 0–14 y/o) (12). The beneficial effect of HU in our study contrasts with the SECURE registry study, where HU showed no effect on hospitalization and COVID-19 severity. However, both single-center (17) and multi-center publications (12, 13) demonstrated that the use of HU was associated with decreased hospitalization rates (17), need for ICU admission (12) or death (13).

Our cohort spans 12 months (March 2020–February 2021), during which time the medications used to treat COVID-19-related respiratory complications evolved significantly. From an early embrace and subsequent rejection of hydroxychloroquine to the recognition that the oral steroids (usually contraindicated in patients with SCD) are beneficial in respiratory failure patients (24) to the FDA approval and increased usage of the antiviral remdesivir (25). ACS was our most common admission diagnosis, present in almost two-thirds of those admitted and treated per our institution’s expert opinion established ACS pathway that included therapies such as antibiotics, inhaled corticosteroids in conjunction with bronchodilators, and supplemental oxygen when indicated. Intravenous or oral corticosteroid use was considered a mainstay of inpatient COVID-19 therapy but was limited in SCD patients given the known association with rebound VOC (26–28). Despite a low rate of dexamethasone usage, inhaled corticosteroids, which we commonly use as part of an established ACS pathway, were used, and may have contributed to low ICU utilization, given their demonstrated association with good outcomes if initiated early in COVID-19 infection (29).

Over 200 million in the United States have been fully vaccinated against COVID-19, with children 5–11 years old recently being approved through Emergency Use Authorization as of October 2021 (30). Given the morbidity observed within our SCD cohort of children and adolescents, COVID-19 vaccination within this age group may prove beneficial in reducing unwanted SCD complications and hospitalizations. Additional end-organ damage resulting from an acute COVID-19 infection in SCD within the 1st two decades of life is unknown but may contribute to future SCD morbidity in an infection that has already exhibited subacute and chronic complications in the form of Long COVID-19 syndrome (31).

At the beginning of the pandemic, there was a poor understanding of the impact of COVID-19 in pediatric patients with SCD. Management guidelines stemmed from early reports conducted primarily in adult patients and evolved with an improved understanding of the disease pathology. Our study was a non-comparative observational study; thus, it was difficult to make comparisons due to confounding by indication. Additionally, our registry captures only those patients that either presented to CNH for care during their COVID-19 infection or provided documentation to their provider if tested out in the community. Patients received standardized therapies based on the severity of their COVID-19 disease, much of which was supportive and not disease-directed. Routine laboratory evaluation encompassing inflammatory and coagulation markers was not universally obtained early in the pandemic. We also have limited information on how the use of blood transfusions and supplemental oxygen compares to other respiratory viral infections and global viral pandemics. While our study cohort included asymptomatic patients with SCD who were tested for various reasons and found to have COVID-19, it is likely there were more patients with SCD at our institution who had COVID-19 and were asymptomatic or had only minimal symptoms. Our study thus likely over-estimates the morbidity associated with COVID-19. Further investigation into the clinical presentation and outcomes with the emergence of variants is needed and may inform long-term sequelae of COVID-19 infection.

## Conclusion

Our study describes one of the largest, SCD single-center experiences of pediatric SCD patients during the COVID-19 pandemic. It will add to a growing body of literature on pediatric SCD and COVID-19 cases describing the impact of specific SCD and COVID-19 related therapies on clinical outcomes. We report similar morbidity patterns (ACS, Pain/VOC) between SCD pediatric and adolescent patients with COVID-19. While morbidity was variable in our cohort, we saw no mortality within our pediatric and adolescent SCD patients with COVID-19 infection similar to observations made by the Quebec registry which spanned 2 years (32). In comparison to previously published adult reports, the absence of mortality may be attributable to lower end-organ damage in our adolescent cohort or the fact that approximately two-thirds of patients were on some form of disease-modifying therapy. Additionally, we saw a wide array of adjunctive therapies (i.e., remdesivir, anticoagulant thromboprophylaxis) added to routine SCD management. Future studies will compare the impact of best practices throughout consecutive years of the pandemic as well as investigate the emergence of long-COVID in patients with SCD.

## Data availability statement

The datasets presented in this article are not readily available because restrictions are provided by our CNH hospital IRB of record. Requests to access the datasets should be directed to AC, acampbell@childrensnational.org.

## Ethics statement

The studies involving human participants were reviewed and approved by Children’s National Hospital Institutional Review Board. Written informed consent to participate in this study was provided by the participants’ legal guardian/next of kin.

## Author contributions

OM, DD, StM, MS, and AC wrote and critically edited the manuscript, designed the study, performed the research, and analyzed the data. RN, AL, BS-B, BM, JB, JW, and SuM critically edited the manuscript and analyzed the data. JB provided statistical analysis. All authors contributed to the article and approved the submitted version.
