# Supplementary material for: Clinical outcomes of children and adolescents with sickle cell disease and COVID-19 infection: A year in review at a metropolitan tertiary pediatric hospital
Source: Front Med (Lausanne). 2023 Feb 17;10:987194. doi: 10.3389/fmed.2023.987194 (PMC9982154; doi:10.3389/fmed.2023.987194)
Supplement: Supplementary file 1 [file Data_Sheet_1.pdf]

Clinical Outcomes of Children and Adolescents with Sickle Cell Disease and COVID-19 Infection: A Year in Review at a Metropolitan Tertiary Pediatric Hospital

Supplementary Tables

Supplementary Table 1: Laboratory Studies in SCD COVID-19 Non-Hospitalized and Hospitalized Patients

Supplementary Table 2: SCD COVID-19 Patients with and without Acute Chest Syndrome

Supplementary Table 3: Baseline Characteristics of SCD COVID-19 Patients Requiring Supplemental Oxygen Therapy

Supplementary Table 4: Laboratory and Radiographic Imaging of SCD COVID-19 Patients Requiring Supplemental Oxygen Therapy

Supplementary Table 5: Demographics and Clinical History - Children vs. Adolescents

**SUPPLEMENTARY TABLE 1: Laboratory Studies in SCD COVID-19 Non-Hospitalized and Hospitalized Patients**							
	**All (N=55)**		**Non-Hospitalized (N=29)**		**Hospitalized (N=26)**		***p-value**
**Hematology labs**	N	Median (IQR)	N	Median (IQR)	N	Median (IQR)	
WBC (10^3/µL)	43	10.4 (7.6-14.9)	17	8.9 (6.7-12.3)	26	12.0 (8.0-15.4)	0.087
Hemoglobin(g/dL)	43	8.4 (7.3-10.2)	17	8.9 (8.0-9.7)	26	8.4 (6.8-10.2)	0.384
Platelet (10^3/µL)	43	277 (190-380)	17	334 (244-389)	26	249 (183-377)	0.253
Absolute Retic. Count (cells/µL)	42	235 (143-328)	16	248 (176-273)	26	222 (143-360)	0.907
**Coagulopathy Labs**							
D-Dimers (mcg/mL)	23	1.7 (0.5-4.7)	2	0.7 (0.5-0.9)	21	1.8 (0.8-4.7)	0.300
Fibrinogen (mg/dL)	19	394 (266-621)	1	270 (NA)	18	417 (266-621)	0.523
PT (seconds)	25	15.1 (14.3-15.8)	2	14.4 (13.9-14.8)	23	15.3 (14.3-15.9)	0.249
INR	23	1.2 (1.1-1.3)	2	1.1 (1.1-1.2)	21	1.2 (1.1-1.3)	0.275
PTT (seconds)	25	33.7 (29.4-37.5)	2	33.5 (29.4-37.5)	23	33.7 (29.1-37.7)	0.999
**Inflammatory Markers**							
CRP (mg/L)	13	2.0 (0.4-6.2)	1	1.2 (NA)	12	2.4 (0.3-6.2)	0.894
ESR (mm/h)	6	22.5 (6.0-42.0)	1	1.3 (NA)	5	35.0 (10.0-42.0)	0.242
Ferritin (ng/mL)	6	416 (45-1265)	4	1358 (645-3434)	5	58 (45-416)	0.270
*p value: Non-Hospitalization vs Hospitalization; CRP=C-reactive protein; ESR=Erythrocyte Sedimentation Rate; Hgb=Hemoglobin; Retic=Reticulocyte; WBC=White Blood Count							

SUPPLEMENTARY TABLE 2: SCD COVID-19 patients with and without Acute Chest Syndrome for hospitalized patients					
	**No Acute Chest (N=12)**		**Acute Chest (N=14)**		***p value**
**Laboratory Studies**	Median (IQR)		Median (IQR)		
WBC (10^3/µL)	8.7 (7.1-12.1)	N=12	14.1 (10.3-16.7)	N=14	0.033
Hemoglobin (g/dL)	9.1 (7.9-10.4)	N=12	7.9 (6.7-9.2)	N=14	0.123
Lowest Hgb (g/dL)	9.6 (7.3-9.9)	N=9	6.8 (6.4-8.8)	N=12	0.064
Platelet (10^3/µL)	191 (135-308)	N=12	282 (247-379)	N=14	0.076
Absolute Retic Count (cells/ µL)	201 (87-328)	N=12	254 (150-414)	N=14	0.247
Creatinine (mg/dL)	0.53 (0.35-0.74)	N=10	0.47 (0.34-0.62)	N=14	0.578
**Coagulopathy Labs**					
D-Dimers (mcg/ml)	0.48 (0.38-0.91)	N=8	3.60 (1.78-5.74)	N=13	<0.001
Fibrinogen (mg/dL)	249 (248-281)	N=5	491 (394-630)	N=13	0.010
PT (seconds)	15.2 (14.3-15.8)	N=10	15.7 (14.5-16.1)	N=13	0.456
INR	1.2 (1.1-1.2)	N=8	1.2 (1.1-1.3)	N=13	0.425
PTT (seconds)	33.4 (27.4-37.7)	N=10	33.8 (31.5-35.9)	N=13	0.975
**Inflammatory Markers**					
CRP (mg/L)	0.4 (0.2-2.0)	N=5	3.5 (1.2-6.3)	N=7	0.330
ESR (mm/h)	8.0 (6.0-10.0)	N=2	42 (35-45)	N=3	0.149
Ferritin (ng/mL)	49 (40-58)	N=2	416 (45-621)	N=3	0.387

*p value: No Acute Chest vs Acute Chest CRP=C-reactive protein; ESR=Erythrocyte Sedimentation Rate; Hgb=Hemoglobin; Retic=Reticulocyte; WBC=White Blood Count

**SUPPLEMENTARY TABLE 3: Baseline Characteristics of SCD COVID-19 Patients Requiring Supplemental Oxygen Therapy for Hospitalized patients**			
	No Oxygen Requirement (N=16)	Oxygen Requirement (N=10)	p value
**Age Median (IQR)**	14 (7-16)	18 (5-20)	0.223
**Sex N (%)**			
Male	8 (50%)	6 (60%)	0.619
Female	8 (50%)	4 (40%)	
**Sickle Cell Genotype N (%)**			
Hgb SS	10 (63%)	9 (90%)	0.237
Hgb SC	2 (13%)	1 (10%)	
Hgb Sβ0 Thal	4 (25%)	0 (0%)	
**Length of Stay (Median, IQR)**	4 (3-6)	6 (5-7)	0.025
**Oxygen Saturation < 95%**	3 (23%)	10 (100%)	0.001
**Maximal Respiratory Support N (%)**			
No Respiratory Support	16 (100%)	0 (0%)	<0.001
Nasal Cannula Oxygen	0 (0%)	7 (70%)	
BiPAP	0 (0%)	3 (30%)	
**Transfusions N (%)**			
Simple Transfusion (Non-Exchange)	6 (38%)	10 (100%)	0.999
Exchange Blood Transfusion	0 (0%)	1 (10%)	0.999
*p value: No oxygen requirement vs oxygen requirements<br>Hgb=Hemoglobin; Sβ0 Thal =Sickle Beta Zero Thalassemia			

**SUPPLEMENTARY TABLE 4: Laboratory and Radiographic Imaging of SCD COVID-19 Patients Requiring Supplemental Oxygen Therapy**					
	**No Oxygen Requirement (N=45)**		**Oxygen Requirement (N=10)**		***p value**
**Laboratory Studies**	**Median (IQR)**		**Median (IQR)**		
WBC (10^3/µL)	8.8 (7.1-12.4)	N=33	14.1 (12.1-17.0)	N=10	0.002
Hemoglobin (g/dL)	8.9 (8.0-10.4)	N=33	7.1 (6.7-8.5)	N=10	0.022
Lowest Hgb (g/dL)	9.2 (6.8-9.9)	N=15	7.1 (6.4-7.9).	N=8	0.066
Platelet (10^3/µL)	270 (183-380)	N=33	307 (248-379)	N=10	0.343
Absolute Retic Count (cells/µL)	228 (134-273)	N=32	357 (183-431)	N=10	0.035
Creatinine (mg/dL)	0.43 (0.36-0.60)	N=28	0.47 (0.34-0.52)	N=10	0.895
**Coagulopathy**					
D-Dimers (mcg/mL)	0.8 (0.5-1.4)	N=13	4.1 (2.8-5.7)	N=10	0.002
Fibrinogen (mg/dL)	270 (249-352)	N=9	601 (439-638)	N=10	0.013
PT (seconds)	14.5 (14.0-15.7)	N=15	16.0 (15.0-16.8)	N=10	0.006
INR	1.1 (1.1-1.2)	N=13	1.3 (1.2-1.4)	N=10	0.005
PTT (seconds)	33.1 (28.8-37.5)	N=15	34.1 (31.5-38.0)	N=10	0.579
**Inflammatory Markers**					
CRP (mg/L)	0.8 (0.2-2.0)	N=6	3.5 (1.2-6.9)	N=7	0.225
ESR (mm/h)	8.0 (3.6-22.5)	N=4	44.0 (42.0-45.0)	N=2	0.105
Ferritin (ng/mL)	416 (40-1,451)	N=7	333 (45-621)	N=2	0.884
**CXR Findings**					
Normal	20 (44%)		1 (10%)		<0.001
Single Infiltrate	2 (5%)		0 (0%)		
Multi-Lobar Infiltrate	4 (9%)		9 (90%)		
Not Completed	20 (44%)		0 (0%)		
*p value: No oxygen requirement vs oxygen requirements<br>CRP=C-reactive protein; ESR=Erythrocyte Sedimentation Rate; Hgb=Hemoglobin; Retic=Reticulocyte; WBC=White Blood Count					

**SUPPLEMENTARY TABLE 5: Demographics and Clinical History - Children vs Adolescents**			
	**Children <18y/o (N=38)**	**Adolescents ≥ 18 y/o (N=17)**	**p value**
**Age (Mean, SD)**	8.3 (5.2)	19.2 (1.2)	
Male	17 (45%)	10 (59%)	0.334
Female	21 (55%)	7 (41%)	
**Sickle Cell Genotype (N, %)**			
Hgb SS	30 (79%)	11 (65%)	0.442
Hgb SC	4 (11%)	4 (24%)	
Hgb Sβ0 Thal	4 (11%)	2 (12%)	
**Disease Modifying Treatments (N, %)**			
Hydroxyurea	25 (66%)	11 (65%)	0.938
Voxelotor	1 (3%)	1 (6%)	0.527
Crizanlizumab	0 (0%)	3 (18%)	
**Chronic Blood Transfusion**	4 (11%)	0 (0%)	0.299
**Healthcare Utilization (N, %)**			
Hospitalized	17 (45%)	9 (53%)	0.573
ED only patients	10 (26%)	4 (24%)	0.999
ICU patients	1 (3%)	2 (12%)	0.223
**Sickle Cell Disease Presentation (N, %)**			
Vaso-occlusive Crisis (any)	12 (32%)	7 (41%)	0.489
Acute Chest Syndrome	7 (18%)	7 (41%)	0.073
*p value: Children vs Adolescents<br>Hgb=hemoglobin; ED= Emergency Department			
